# Expression Pattern of Cathelicidins in Dairy Cows During Endometritis and Role of Bovine Endometrial Epithelial Cells in Production of Cathelicidins

**DOI:** 10.3389/fvets.2021.675669

**Published:** 2021-09-20

**Authors:** Yajuan Li, Xiaoyu Ma, Jie Yang, Xiaohu Wu, Zuoting Yan, Baoxiang He

**Affiliations:** ^1^College of Animal Science and Technology, Guangxi University, Nanning, China; ^2^Key Laboratory of Veterinary Pharmaceutical Development of Ministry of Agriculture, Lanzhou Institute of Husbandry and Pharmaceutical Sciences of Chinese Academy of Agricultural Sciences, Lanzhou, China

**Keywords:** dairy cow, endometritis, cathelicidin, bovine endometrial epithelial cell, *E. coli*

## Abstract

Endometritis is a common bacterial disease of dairy cows. Cathelicidins are host-defense peptides that play important roles in clearance of bacteria. However, the expression pattern of these peptides during endometritis is still unclear. We hypothesize that the levels of bovine cathelicidins increased during endometritis. This study was to investigate the changes of bovine cathelicidins during endometritis. Forty-four post-partum cows (28–35 days after calving) involved in this study were grouped according to the character of vaginal discharge (VD) into three groups. These were (1) cows with clear fluid (*n* = 8, healthy cows group, N); (2) cows with VD containing <50% off-white mucopurulent material (*n* = 20, moderate endometritis cows, M); (3) cows with VD containing > 50% yellow or white purulent material (*n* = 16, severe endometritis cows, S). The blood, VD, and endometrial biopsies samples were collected from each cow to assess the levels of cathelicidin 1–7. Furthermore, bovine endometrial epithelial cells (BEECs) were stimulated with different concentration of *Escherichia coli* (2 × 10^6^ and 2 × 10^7^ CFU/mL) to detect the cellular source of cathelicidins. Quantitative real-time PCR (RT-qPCR) was used to detect the relative mRNA expression of cathelicidins, and enzyme-linked immune sorbent assay (ELISA) method were used to measure the protein levels. The mRNA and protein levels of cathelicidin 1–7 significantly increased during bovine endometritis (both moderate and severe endometritis), while samples from severe cases showed lower levels of cathelicidins compared to moderate cases. BEECs can express cathelicidin 1–7, and *E. coli* triggered the release of these proteins. High concentration of *E. coli* decreased the mRNA and protein levels of cathelicidins. Taken together, our results supported that cathelicidins are released as host defense molecules against the bacteria during bovine endometritis, and BEECs play an active role in expression and production of cathelicidins.

## Introduction

Endometritis is one of the major bacterial diseases affecting reproductive performance of dairy cows by lengthening the calving interval, increasing number of services per conception and reducing calving rates (1). It also leads to significant economic loss to dairy industry due to reducing low milk production, increasing treatment cost, and poor fertility (2, 3). Endometritis is reported to mainly result from Gram-negative bacterial infection of the endometrium, such as *Escherichia coli* and *Trueperella pyogenes* (4, 5). The post-parturient invasion and growth of bacteria caused persistence inflammation of endometrium in dairy cows.

Cathelicidin is one of the major classes of mammalian host defense protein which is the crucial component of innate immune system (6). It exhibits multiple functions which includes direct anti-microbial activity, chemotaxis and regulatory function of inflammation (6–8). Ten cathelicidin genes including cathelicidin 1–7, Bac4, Bac2A, and IDR-1018 has been reported in cow (8), nevertheless, humans and mice only have one cathelicidin gene (9). Hence, these proteins are believed to play a crucial role in immunity of dairy cows. These proteins are confirmed to be released into milk during mastitis (10), and the expression of cathelicidin is also detected in mammary tissues (11). However, the expression pattern of cathelicidins during bovine endometritis still remains unknown.

It is well-known that cathelicidins are stored as intracellular granules in polymorphonuclear neutrophils (PMNs) which are recalled into the local tissues when infection occurs (12). In addition to PMNs, epithelial cell is also the important source of cathelicidins (13–15). In dairy cows, mammary epithelial cell is confirmed to produce and release cathelicidins (16). The role of bovine endometrial epithelial cells in expression and release of cathelicidins is not clearly defined. We assumed that cathelicidins also can be produced by endometrial epithelium cells to provide the first line of defense microbial invasion in bovine endometrium.

The objectives of this study were to investigate the role of bovine cathelicidins during endometritis, and detect the expression and production of cathelicidins in BEECs stimulated with *E. coli*.

## Materials and Methods

### Vaginal Discharge Collection and Grouping

The samples and clinical data were collected from Gansu Holstein Dairy Cow Breeding Center (Lanzhou, China). All cows were all housed, and the average annual temperature of Lanzhou city is −12–28°C. The inclusion criteria for the dairy cows in the present study were: (1) cows aged 3–5 years and 2–3 parities; (2) cows on 28–35 days post-partum; (3) cows without any signs of systemic symptoms during the post-partum period. The diagnostic criterion of endometritis was the presence of a purulent vaginal discharge, and the cows with clear vaginal discharge were considered as normal cows (1). Total of 125 post-partum cows were included in our screening scope. And forty-four post-parturient cows were adopted for further study based on the above standards. Vaginal discharge (VD) samples were collected using metricheck device (Simcro Limited, Mew Zealand). Briefly, the tail was lifted and perineum was cleaned with dry paper to remove fecal material and then wiped with 75% ethanol. Metricheck device was disinfected with 75% ethanol and then gently introduced into the vagina, the device was withdrawn and the discharge was collected in the cup. The cup was washed with saline and disinfected with 75% ethanol before the next collection. VD samples were transported to the laboratory on ice within 4–6 h of collection at the temperatures of 4°C.

Cows were grouped into normal cows group (N), moderate endometritis group (M), and severe endometritis group (S) according to the properties of vaginal discharge. The vaginal mucus was assessed by one person for color, proportion and volume of pus, and the character of the three groups assigned as follow: N, clear or translucent vaginal discharge; M, the vaginal discharge containing <50% off-white mucopurulent material; S, vaginal discharge containing > 50% yellow or white purulent material (17). The present study was approved by the Institutional Animal Care and Use Committee of Lanzhou Institute of Husbandry and Pharmaceutical Sciences of Chinese Academy of Agricultural Sciences (SYXK-2014-0002).

### Vaginal Discharge Processing

In the laboratory, pre-treatment of VD was modified according to method described for bovine vaginal discharge (18). Briefly, the discharge weighing ~1 g was taken from each sample and treated with sterile PBS (Gibco, Australia) containing 2 μg/mL protease inhibitor (Solarbio, China) at ratio 4:1 (v/w). Each sample was vortexed for 30 s and then rocked for 45 min at room temperature. Then the samples were centrifuged at 12,000 × g for 30 min at 4°C and the supernatant was collected for subsequent test. Bicinchoninic acid assay (BCA Protein Assay Kit Takara, Japan) was used to measure total protein level in the discharge samples, and the samples with higher total protein were diluted with PBS. Finally, total protein content in each sample is same. Then the working samples were stored at −80°C until further analysis.

### Tissue and Blood Samples Collection

Endometrial biopsies were performed using a biopsy forceps. Briefly, biopsy forceps (purchased and customized from online shops of the Taobao Mall, https://m.tb.cn/h.4zmpAhw?sm=fd2fa2, Supplementary Figure 1) was disinfected with 75% ethanol and introduced into the vagina. The tip of the forceps was guided into the horn of uterus through manipulation per rectum by an experienced veterinary. The biopsy was obtained approximately halfway between the cervix and utero-tubal junction carefully. A 3 × 3 mm tissue section was obtained using this forceps. Collected biopsy was placed into petri dish containing normal saline. Every tissue sample was divided into two pieces and immediately preserved in either liquid nitrogen for total RNA extraction or 10% formalin solution for histopathology examination using routine hematoxylin and eosin (HE) staining. Then, four-micrometer-thick sections cut from the fixed specimens were mounted on slides. Histologically, endometritis is characterized by some disruption of surface epithelium, infiltration with inflammatory cells, vascular congestion, and stromal edema and by varying degrees of lymphocyte and plasma cell accumulation in the superficial layers (19, 20). Slides were observed under microscope (Olympus, BX43), and assessed according to the histological features. The blood samples of tissues-collected cows were collected from caudal vein using vacuum tubes without anticoagulant and immediately stored on ice. Serum samples were harvested from the blood within 2 h, and were stored at −80°C until analysis.

### Cytological Smear Preparation

Duplicate cytology smears were prepared immediately after the VD samples were collected. The slides were air-dried and stained using Diff-Quick staining protocol (Solarbio, China). Smears were evaluated microscopically and proportions of neutrophils were recorded. All cells were counted and the percentages of PMNs were calculated from average of 200 cells (18).

### Cells Isolation and Culture

Bovine endometrial epithelial cells (BEECs) were isolated from a healthy uterus of a 6-month old dairy cow according to our previous study (19). Briefly, a healthy uterus was brought to laboratory within sterile PBS (pH 7.2) containing penicillin (100 μg/mL) and streptomycin (100 U/mL). Endometrium from uterine horn was cut off into 2–3 mm long pieces, washed in PBS (pH 7.2) twice, and then uterine tissue was digested with 1% collagenase I (Sigma, USA) diluted in DMEM/F12 (HyClone, USA) for 6 h. The digested endometrium was scraped using a sterile cell scraper, and scraped materials was collected and washed in PBS (pH 7.2). Then, the collected materials were centrifuged at 100 g for 5 min to collect cell suspension. Trypan Blue stain was used to estimate cells viability. Cells were cultured in DMEM/F12 with 10% fetal bovine serum (Gibco, Australia) and penicillin (100 μg/mL) and streptomycin (100 U/mL) at 37°C with a 5% CO_2_ and 95% sterile air when the viability >95%. The medium was changed every 3 days until the cells reached ~90% confluence.

Considering that the cultures were inevitably mixed by some stromal cells, which were removed according to the different sensitivity of epithelial cells and stromal cells to trypsin, and time-different digestion was conducted to obtain purified epithelial cells. The expression of epithelial-specific cytokeratin was adopted to estimate the purification of epithelial cells through using a mouse monoclonal antibody (Abcam, ab49779) and goat anti-mouse IgG-FITC (Abcam, ab6785).

### BEECs Stimulated With *E. coli*

BEECs growing in logarithmic phase were inoculated into a six-well plate at the concentration of 2 × 10^5^ cells/well and incubated for 24 h, then treated with *E. coli* (ATCC 25922) at 2 × 10^6^, 2 × 10^7^, 2 × 10^8^, 2 × 10^9^, 2 × 10^10^ CFU/mL for 2 h, respectively. And the cell viability was estimated using a CCK-8 kit (Biosharp, China). Cell supernatant was collected for the detection of cathelicidin protein level.

### Detecting the mRNA Expression of Cathelicidins Using RT-qPCR Method

Total RNA was extracted from BEECs and endometrial biopsy tissues using RNAiso Plus (Takara, Japan) according to the manufacture's protocol, then total of 1,000 ng RNA was used for reverse transcription by PrimeScript RT reagent Kit with gDNA Eraser (Takara, Japan). RT-qPCR was performed using SYBR Premix Ex TaqII (Takara, Japan) on an iQ5 thermal cycler (BioRad, US). Each 20 μL reaction was set up with 10 μL 2 × SYBR Premix Ex Taq, 0.4 μL of each primer (forward and reverse, 10 μM), 2 μL cDNA and 7.2 μL RNAse free water. All samples were run in duplicate. The reactions were incubated though an initial activation and denaturation step at 95°C for 30 s, followed by 40 cycle of 5 s at 95°C, 10 s at 60°C, and 5 s at 72°C. A melt curve analysis was performed between 60 and 95°C in 0.5°C increments to confirm the identity of the amplicons. The fold change of mRNA expression to β-actin was calculated using the 2^−ΔΔCt^ method (21). The applied primers were detailed in Table 1.

**Table 1:** Primer pairs used for RT-qPCR.

**Gene name**	**ID**	**Sequence**	**Production length**	**Temperature**
β-actin	NM_173979.3	F-CTCTTCCAGCCTTCCTTCCT	179	61
		R-GGGCAGTGATCTCTTTCTGC		
Cathelicidin 1	NM_174825.1	F-CCAGGTCAGGGGTAACTTCG	249	
		R-AAACCCTTAGGACTCTGCTGG		59
Cathelicidin 2	NM_174826.3	F-GGAGCTAGACCCTACACCCA	257	60
		R-GATAGAACGGCGGACGGATT		
Cathelicidin 3	NM_174001.2	F-ATGAGCGGTCCTCAGAAGC	187	61
		R-TCCCCACACACTGTTTCACC?		
Cathelicidin 4	NM_174827.2	F-AGACCCACCTCCCAAGGATAA	169	60
		R-CACTGTTTCACCCGCCCTTTC		
Cathelicidin 5	NM_174510.3	F- ACTTCGAAGCCTGGGTAGGA	183	60
		R- GGGCCCACAATTCACCCAA		
Cathelicidin 6	NM_174832.3	F-ACTCTGGATGCGGTGAAAGG	254	62
		R-AGGGAGGACACACACAGGAT		
Cathelicidin 7	NM_17832.11	F-CAAGGAGAATGGGCTGGTGA	185	61
		R-TCTGCTGACCTCTCCGGATT		

### Measurement of Cathelicidins in Vaginal Discharge and Serum Samples

The contents of cathelicidin 1–7 protein were detected using ELISA kit (MLBIO, China) according to the instruction of the manufacture. MULTISKAN MK3 (Thermo, USA) was used to read plate. The use of analyte optical densities and their corresponding expected concentrations to calculate the observed concentration using a 4-parameter logistic regression analysis.

### Statistical Analysis

Statistical analysis and graphs were performed using GraphPad prism 8 software (GraphPad Software, Inc. USA). One-way ANOVA analysis was used to compare the results between N group and M/S group, or between different treated cells. All data were presented as mean ± SD. Statistical significance was considered at *P* < 0.05.

## Results

### Vaginal Discharge Type Analysis

**Figure 1:**
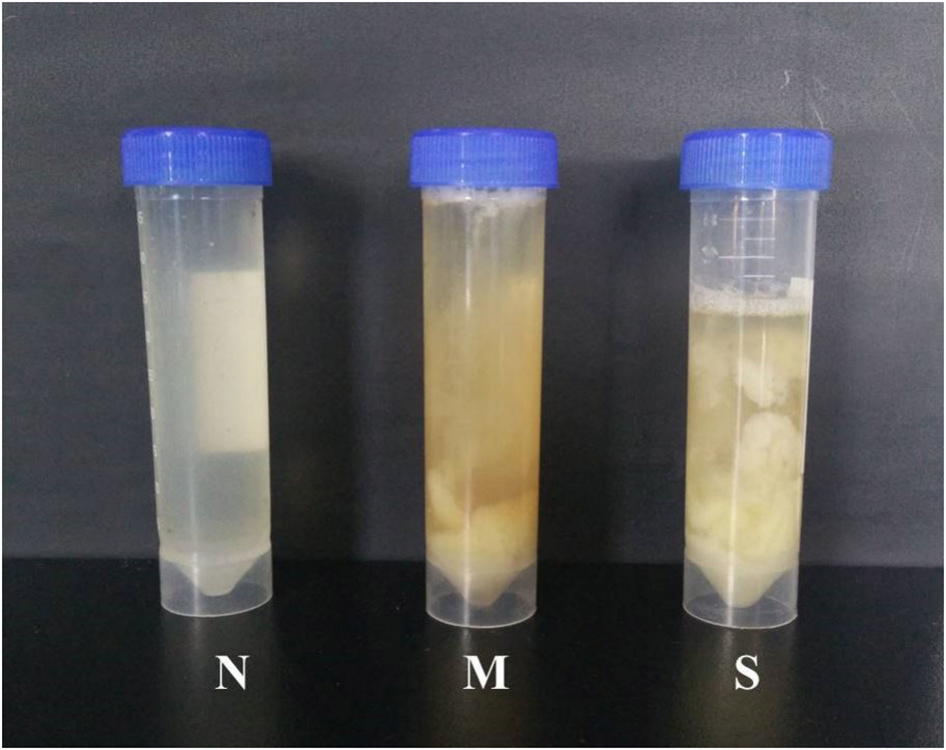
Typical samples of vaginal discharge character: N means clean vaginal discharge from normal cows; M means discharge containing <50% white or off-white pus from dairy cows with moderate endometritis; S means discharge containing > 50% white or yellow purulent material.

Clinical examination of forty-four vaginal discharge samples revealed that 8 cows were healthy cows (normal group, N, *n* = 8), 20 cows were diagnosed with moderate endometritis (moderate endometritis group, M, *n* = 20), and 16 cows were diagnosed with severe endometritis (severe endometritis group, S, *n* = 16). The typical discharge characteristic in each group was shown in Figure 1.

### VD Cytology

**Figure 2:**
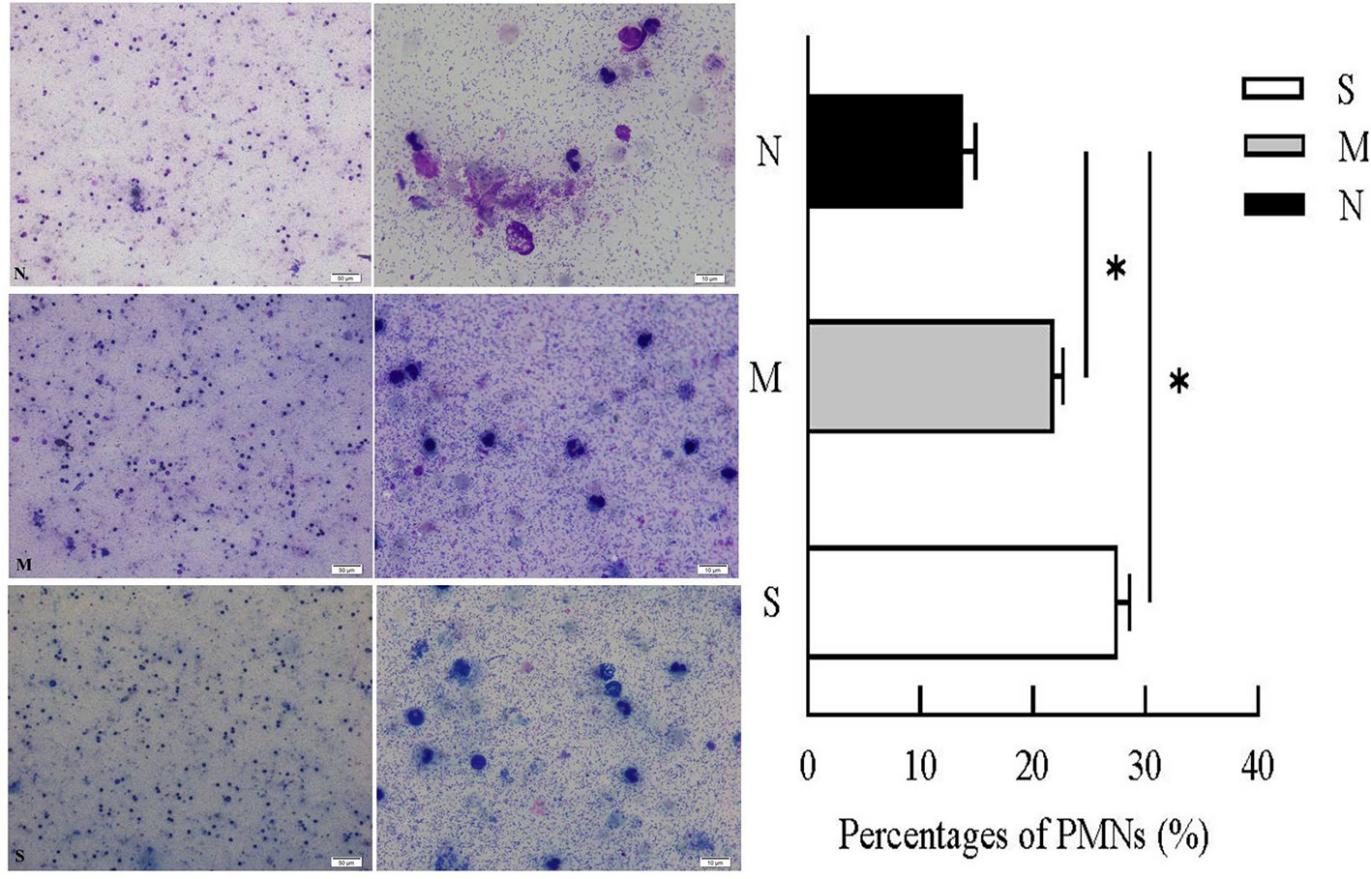
Percentages of PMNs were calculated from VD samples in N, M, and S group. N, M, and S represents the samples from normal cows, cows with mild endometritis and severe endometritis, respectively. All cells were counted and the percentages of PMNs were calculated from average of 200 cells. Representative image of cytological smears from N, M, and S group were presented. *P < 0.5, compared with the N group.

Cytological examination revealed recruited PMNs in VD collected 28–35 days post-partum (Figure 2). PMN counts of VD were <15% (13.76 ± 0.91%) in normal cows. In cows with endometritis, percentages of PMNs were significantly higher than in normal cows, the average PMNs% in cows with moderate endometritis and severe endometritis was 21.84 ± 0.69% and 27 ± 0.86%, respectively.

### Histopathologic Examination of Different Severity of Endometritis

**Figure 3:**
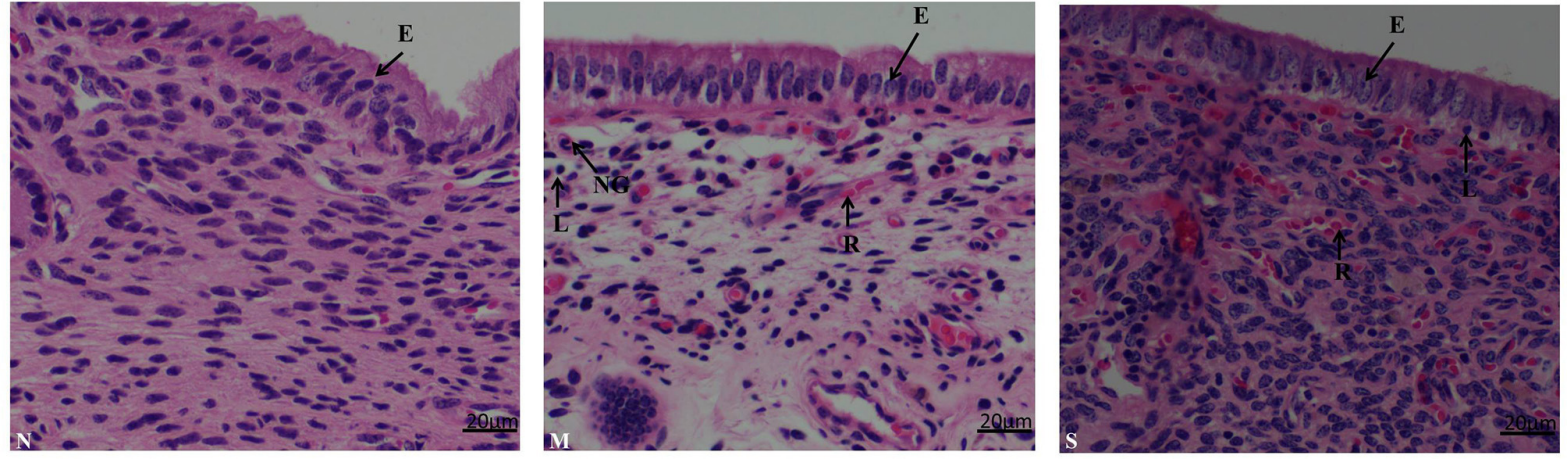
Histopathological characterization in endometrium biopsy samples from postpartum cows. N, M, and S represents the samples from normal cows, cows with moderate endometritis and severe endometritis, respectively. Cells labeled as E, epithelial cell; N, neutrophil; L, lymphocyte; P, plasmocyte; R, red blood cell, NG, neutrophile granulocyte.

As shown in (Figure 3) the endometrial biopsy from healthy cows showed scanty presence of red blood cells (N); the biopsy from mild endometritis cows characterized by the abundant infiltration of lymphocytes, neutrophile granulocyte, and plasma cells, and also a few neutrophils and blood cells, and significant swelling of epithelial cells (M); severe endometritis (S) exhibited a degenerative necrosis of the epithelial cells with nuclear condensation, and damage of the mucosal layer; there was congestion of the capillaries with significant infiltration of red blood cells, granulocytes and lymphocytes, resulting in a general increase in cell density.

### The Protein Level of Cathelicidins in VD and Serum Samples

**Figure 4:**
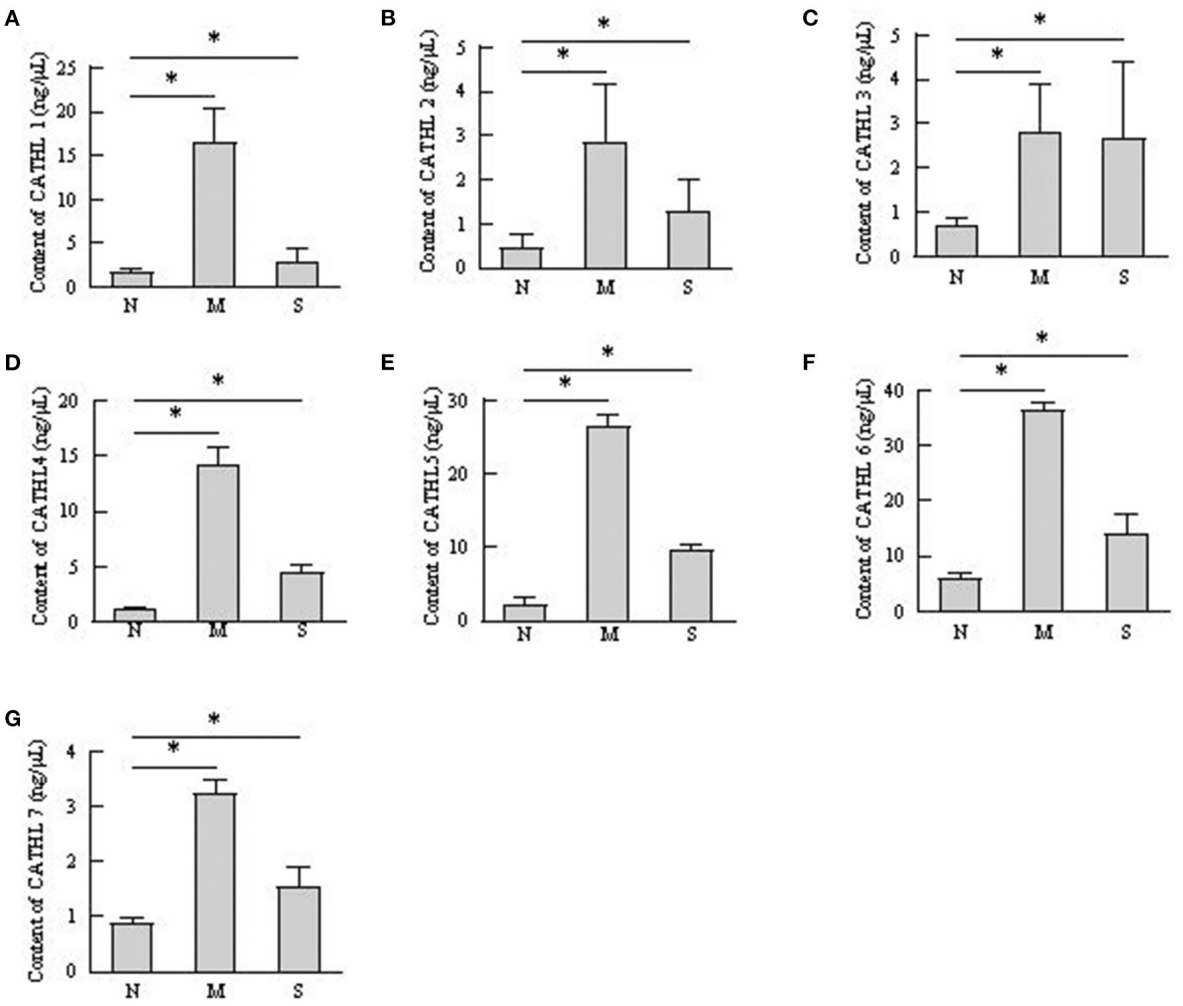
Contents of cathelicidin 1–7 in vaginal discharge detected by ELISA. Vaginal discharge samples were collected from N, M, and S represents the samples from normal cows (*n* = 8), cows with moderate endometritis (*n* = 20) and severe endometritis (*n* = 16), respectively. *P < 0.5, compared with the N group; **(A–G)** Cathelicidin 1–Cathelicidin 7.

**Figure 5:**
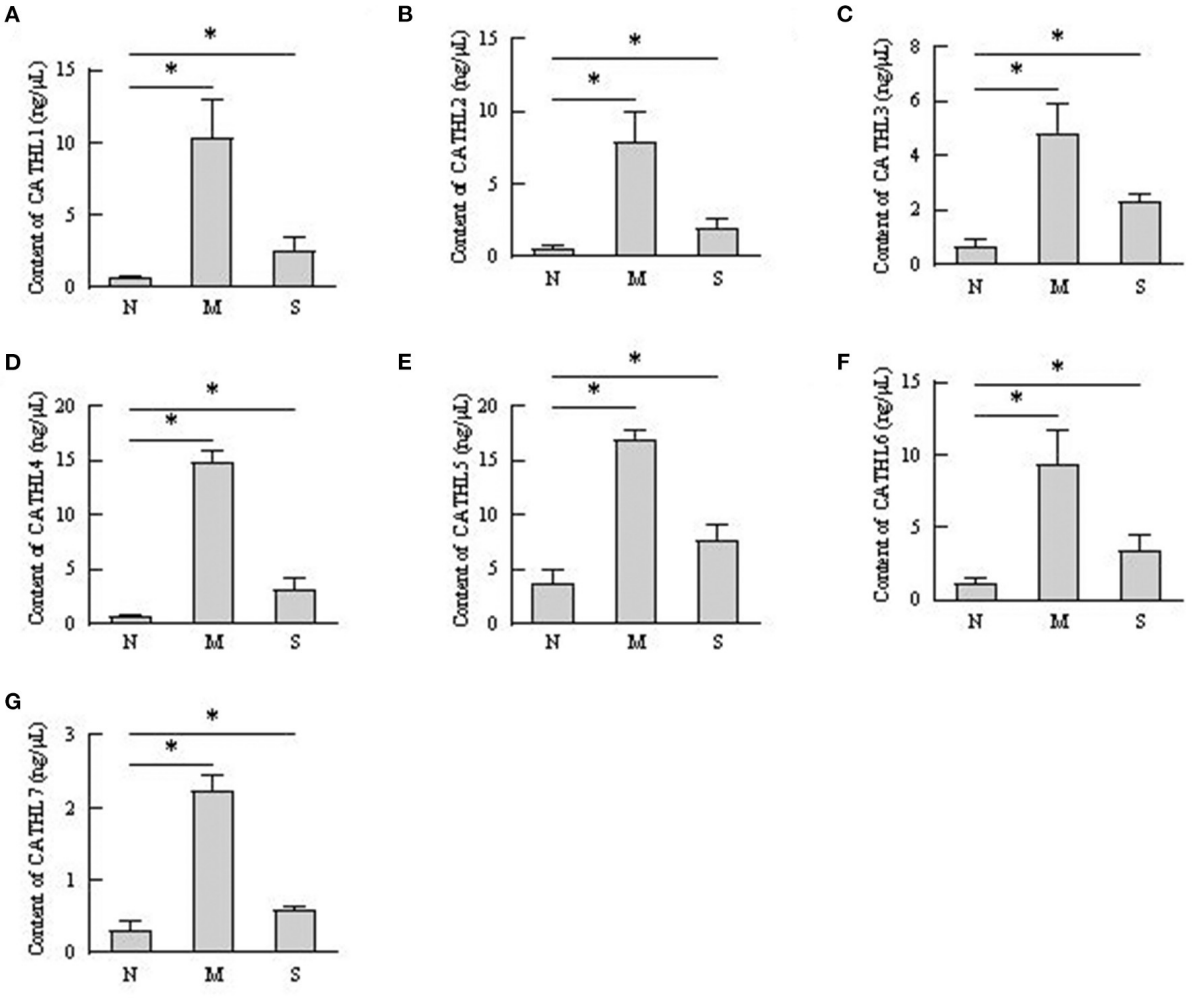
Contents of cathelicidin 1–7 in serum detected by ELISA. N, M, and S represents the samples from healthy cows, cows with moderate endometritis and severe endometritis, respectively. *P < 0.5, compared with the N group. **(A–G)** Cathelicidin 1–Cathelicidin 7.

The protein levels of cathelicidins were estimated per mL of every discharge sample or serum sample. As shown in Figure 4, cathelicidin 1–7 protein in VD samples significantly increased in dairy cows with endometritis compared with normal cows regardless of the severity, however, those proteins were lower in S group than in M group. As shown in Figure 5, cathelicidin 1–7 can be detected in serum, and all measured cathelicidins showed similar alteration with VD samples.

### mRNA Expression of Cathelicidins in Endometrium Tissues

**Figure 6:**
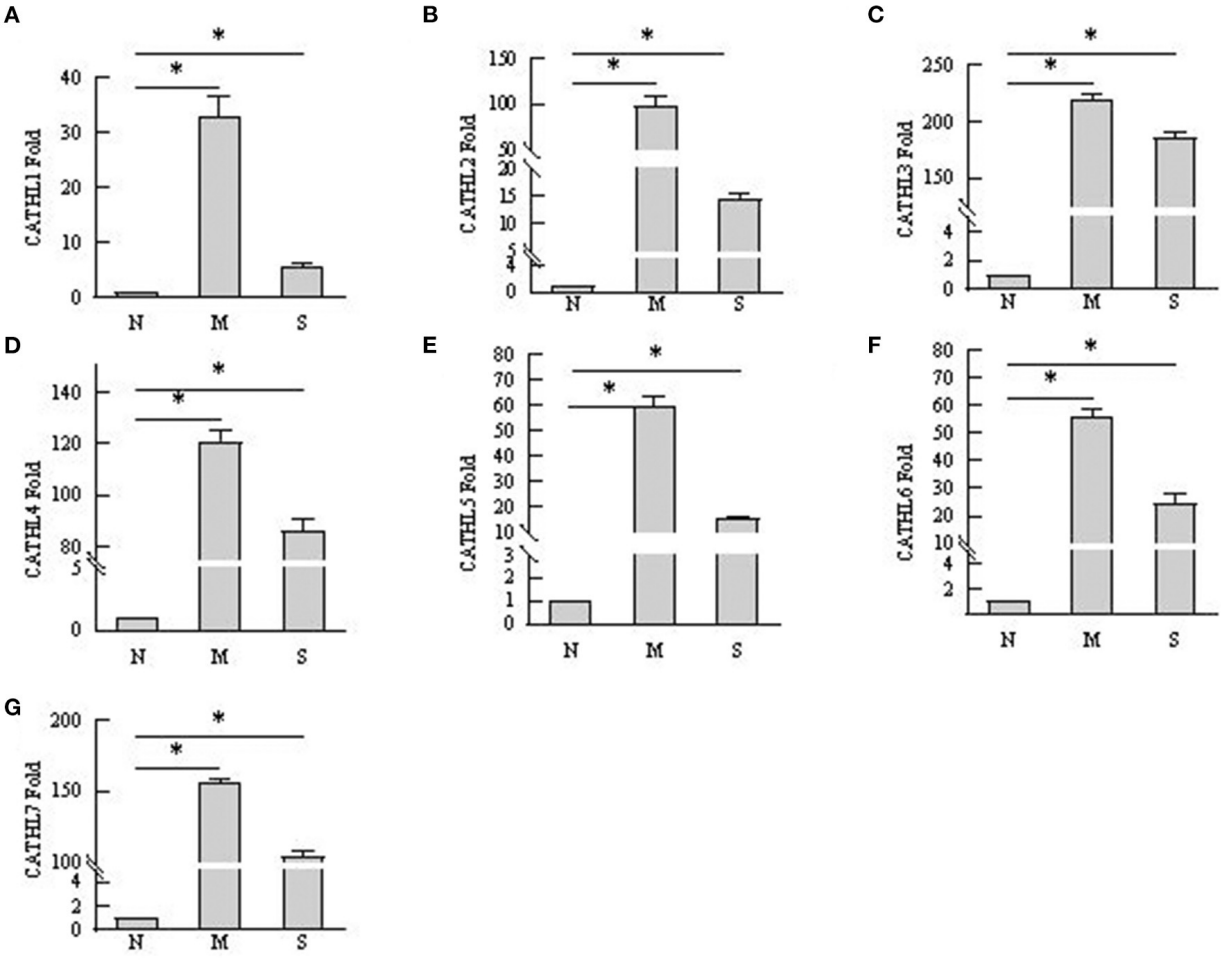
mRNA expression changes of cathelicidin 1–7 in endometrium tissues. N, M, and S represents the samples from normal cows, cows with mild endometritis and severe endometritis, respectively. *P < 0.5, compared with the N group. **(A–G)** Cathelicidin 1–Cathelicidin 7.

As shown in Figure 6, the results indicated that the mRNA expression of cathelicidin 1–7 can be detected in endometrial samples, and the mRNA level of cathelicidin 1–7 increased in tissues with endometritis regardless the severity. However, the expression of the detected cathelicidins decreased in S group than in M group.

### The Expression and Release of Cathelicidins in BEECs Treated With *E. coli*

**Figure 7:**
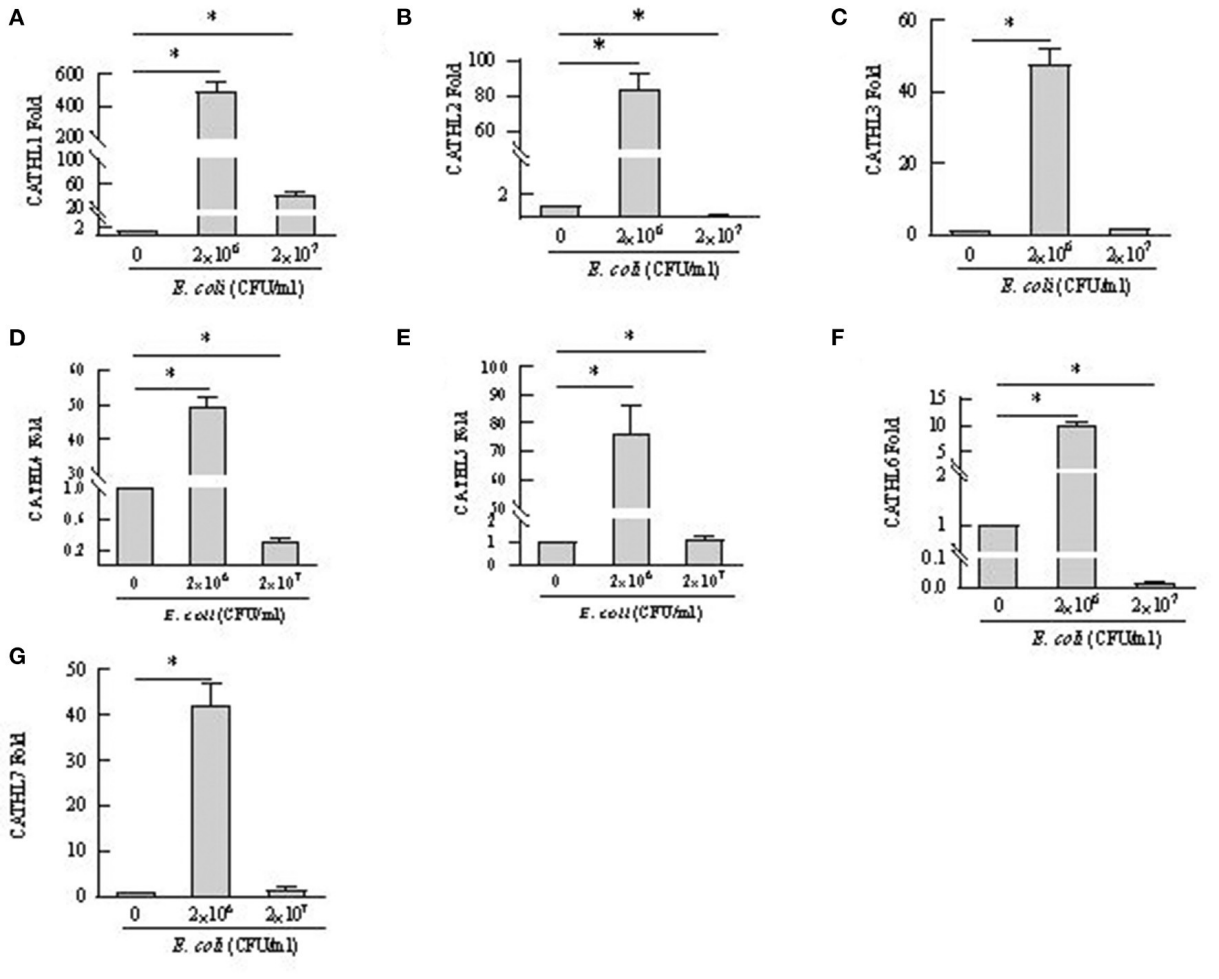
mRNA expression changes of cathelicidin 1–7 in bovine endometrial epithelial cells treated with *E. coli*. *P < 0.5, compared with the untreated cells. **(A–G)** Cathelicidin 1–Cathelicidin 7.

**Figure 8:**
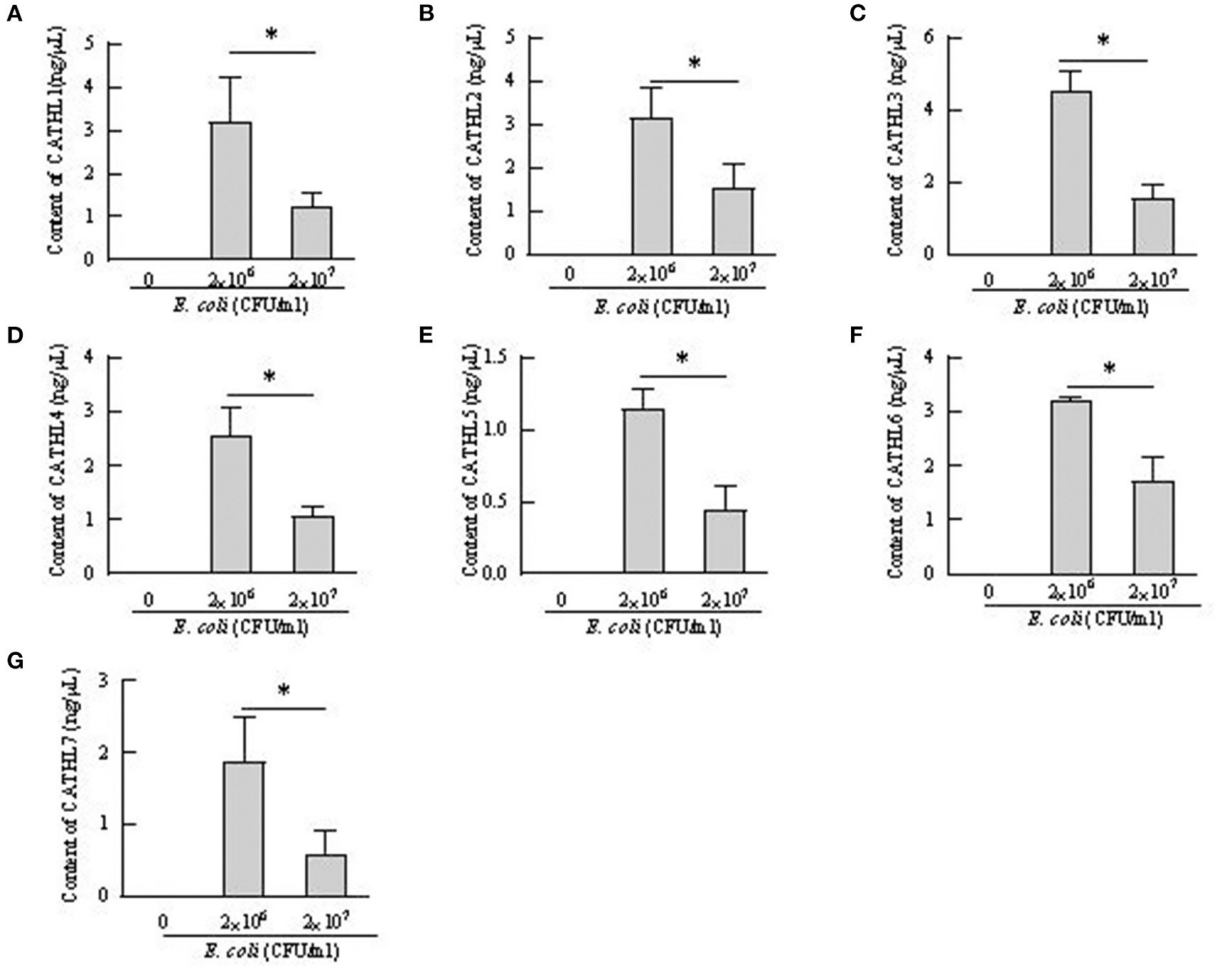
Contents of cathelicidin 1–7 in cell supernatant from bovine endometrial epithelial cells treated with *E. coli*. *P < 0.5, compared with the untreated cells. **(A–G)** Cathelicidin 1–Cathelicidin 7.

Apoptosis of more than 80% of BEECs were observed through the microscope treated with more than 2 × 10^8^ CFU/mL *E. coli*. 2 × 10^6^ CFU/mL and 2 × 10^7^ CFU *E. coli* were used to stimulate BEECs as inflammatory model. Figure 7 showed that the expression of cathelicindin 1–7 significantly increased in BEECs treated with 2 × 10^6^ CFU/mL *E. coli*, but 2 × 10^7^ CFU *E. coli* leads to repression of the expression. Protein levels of cathelicidins were undetectable in the cell supernatant from cells without *E. coli*, and *E. coli* triggered the release of cathelicidns (Figure 8). Protein levels triggered by 2 × 10^6^ CFU/mL *E. coli* were higher than 2 × 10^7^ CFU/mL *E. coli*.

## Discussion

Uterus is normally sterile throughout pregnancy and exposed to many bacteria during post-partum period. Infection of endometrium with Gram-negative bacteria is the major pathogenesis of endometritis in cattle (22). Acting as parts of the innate host defense, cathelicidin is directly involved in pathogen clearing to providing a first line of defense against invading microbes (23). Markedly ascending cathelicidins in the milk of cows with mastitis has been observed (10), less is known about their roles during bovine endometritis. The present study firstly investigated the roles of the cathelicidins in cattle with endometritis. Levels of cathelicidins increased in vaginal discharge, serum, and endometrial biopsies samples from cows with endometritis, regardless of the severity of endometritis. These findings accords with the general reports that increased levels of cathelicidins are observed in a range of inflammatory conditions (8, 9, 24). The results demonstrated that cathelicidins play distinct roles in defensing against bacteria in uterus. Additionally, as shown in Figure 5, the concentrations of cathelicidins in serum of cows with endometritis were increased, which suggested that cows with endometritis showed systemic inflammatory response (24).

Several studies confirmed that cathelicidins increased in inflammatory disease caused by bacterial infection (25). Nevertheless, there was no report about whether the levels of cathelicidins increased in the rising severity of the disease. We observed that the levels of cathelicidins were higher in severe endometritis samples than in samples from normal cows, but their levels were lower than in moderate endometritis samples (Figures 4–6), thus indicating that the expression and production of cathelicidins were weakened in severe cases. We found for the first time that cathelicidins decreased in severe endometritis.

Extensive PMNs were recruited into uterus from the circulation to respond against the invading pathogens (26), and these immune cells can secrete cytokines, chemokines, and antimicrobial peptides at the site of infection as part of the inflammatory responses to assist in clearance of the infection (27). Cathelicidins can be stored in polymorphonuclear neutrophils (PMNs) as secondary granules and then released upon bacterial infection (28), and they always show good correlation with PMN (8). However, in our study, vaginal discharge samples with high percentage of PMN showed lower concentration of cathelicidins (Figures 2–4). We speculated that PMN is not the only cellular resource of cathelicidins in uterus. With the exception of PMN, cathelicidin can also be expressed and produced by epithelial cells (29). Cubeddu et al. (16) found that in areas showing inflammatory lesions, only residual intact mastitis epithelial cells can express cathelicidin. Tomasinsig et al. (25) reported that the protein levels of cathelicidins showed good positive correlation with the percentage of PMN in uterine cyto-brush samples of post-partum cows, which is contradictory to our findings. In this study, we selected the cows at 28–35 day post-partum, whereas Ledgard et al. (27) chose the cows at 15 day post-partum. Persistent infection could cause endometrial damage. Histopathological signs of severe endometritis (Figure 3) showed epithelial necrosis of endometrial tissue. Based on these findings, we assumed that dairy cows can produce endometrial cathelicidins.

**Figure 9:**
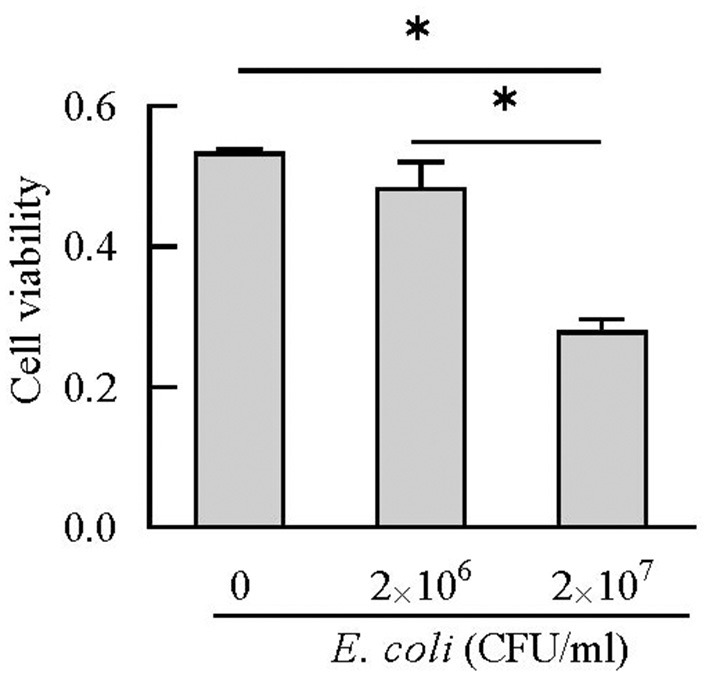
Cell viability was estimated using CCK-8 kit. *P < 0.5, compared with the untreated cells.

BEECs can express and produce chemokines and acute phase proteins to defense the invading pathogens (30, 31), while the role of BEECs in the expression and production of cathelicidins is still unclear. Our results indicted that BEECs can express cathelicidin, but the proteins were undetectable in the cell supernatant samples without bacteria. Moreover, low concentration of *E. coli* (2 × 10^6^ CFU/mL) enhanced their mRNA expression and triggered the release of cathelicidins (Figures 7, 8). Similarly, Chromek et al. (32) detected the mRNA expression of cathelicidin in renal epithelial cells, and the release of cathelicidin was detected upon contact with bacteria. Nevertheless, we observed a decrease in the mRNA expression and protein levels of cathelicidins in BEECs upon high concentration of *E. coli* (2 × 10^7^ CFU/mL) (Figures 7, 8). And BEECs treated with 2 × 10^7^ *E. coli* showed significant decreased cell viability (Figure 9). In the *in vivo* mastitis model, cathelicidin only can be expressed in residual epithelial cells of heavily inflamed area (32). The damage of cells may restrain the expression and release of cathelicidins.

## Conclusion

In conclusion, dairy cows can produce endometrial cathelicidins, and the expression and production of these cathelicidins increased during endometritis. Cathelicidins production decrease in sever endometritis. BEECs play an active role in expression and production of cathelicidins to defend against bacterial infection.

## Data Availability Statement

The raw data supporting the conclusions of this article will be made available by the authors, without undue reservation.

## Ethics Statement

The animal study was reviewed and approved by Institutional Animal Care and Use Committee of Lanzhou Institute of Husbandry and Pharmaceutical Sciences of Chinese Academy of Agricultural Sciences (SYXK-2014-0002).

## Author Contributions

ZY and BH: conceptualization. YL and XM: methodology. YL and JY: data curation. YL, XW, and JY: validation. YL: writing—original draft. ZY and BH: writing—review and editing and funding acquisition. All authors have read and agreed to the published version of the manuscript.

## Funding

This research was funded by National Key R&D Program of China (No. 2017YFD0502201), Science and Technology Innovation Project (No. CAAS-ASTIP-2014-LIHPS-03), and Key research and development plan of Gansu province (20YF8NA029).

## Conflict of Interest

The authors declare that the research was conducted in the absence of any commercial or financial relationships that could be construed as a potential conflict of interest.

## Publisher's Note

All claims expressed in this article are solely those of the authors and do not necessarily represent those of their affiliated organizations, or those of the publisher, the editors and the reviewers. Any product that may be evaluated in this article, or claim that may be made by its manufacturer, is not guaranteed or endorsed by the publisher.
