# Supplementary figures and images for: Expression Pattern of Cathelicidins in Dairy Cows During Endometritis and Role of Bovine Endometrial Epithelial Cells in Production of Cathelicidins
Source: Front Vet Sci. 2021 Sep 20;8:675669. doi: 10.3389/fvets.2021.675669 (PMC8489660; doi:10.3389/fvets.2021.675669)

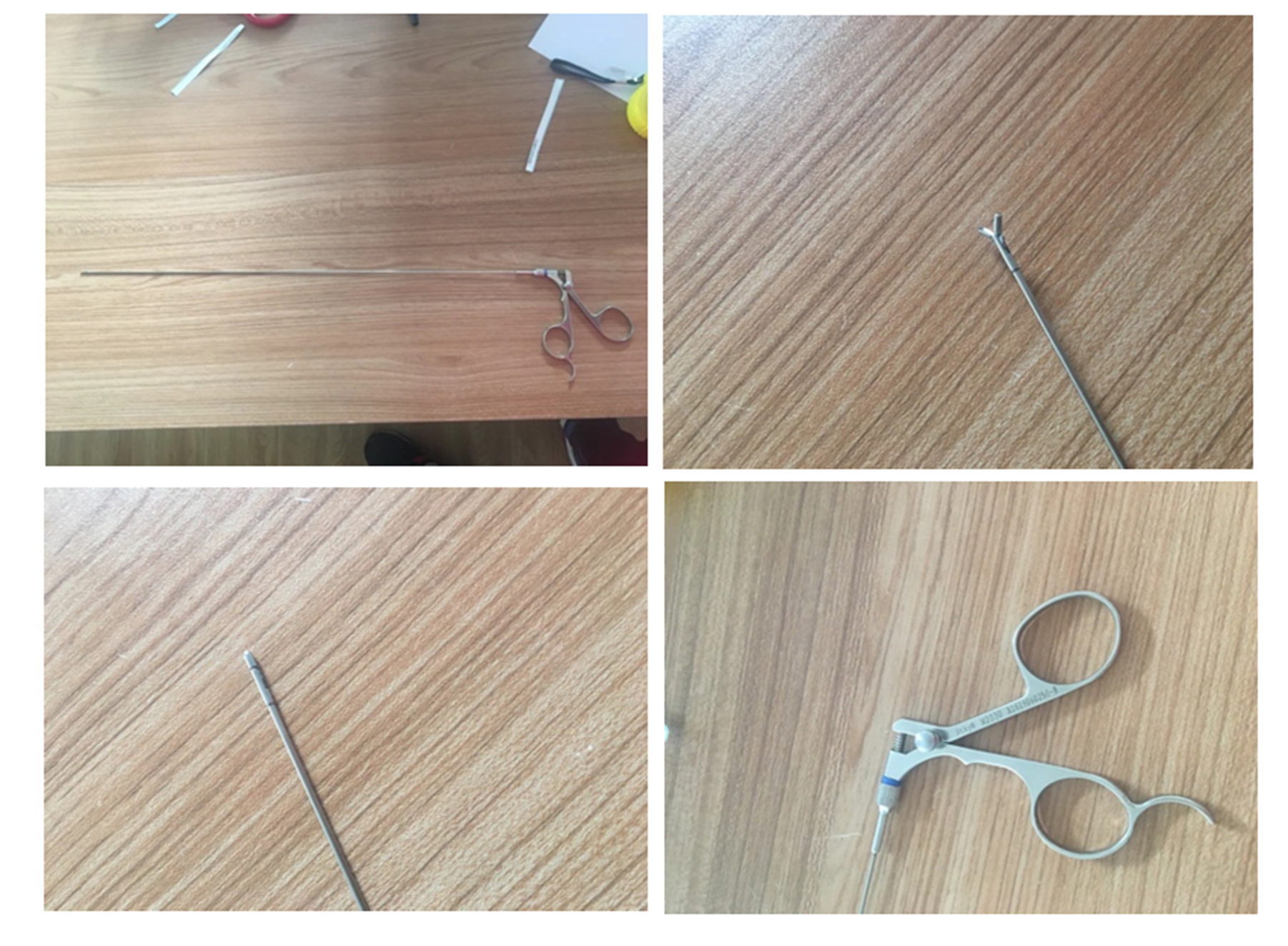

Supplement: Supplementary file 1 [file Image_1.PNG]
